# Clathrin-Mediated Albumin Clearance in Alveolar Epithelial Cells of Murine Precision-Cut Lung Slices

**DOI:** 10.3390/ijms24032644

**Published:** 2023-01-31

**Authors:** Vitalii Kryvenko, Andrés Alberro-Brage, Athanasios Fysikopoulos, Miriam Wessendorf, Khodr Tello, Rory E. Morty, Susanne Herold, Werner Seeger, Christos Samakovlis, István Vadász

**Affiliations:** 1 Department of Internal Medicine, Justus Liebig University, Universities of Giessen and Marburg Lung Center (UGMLC), 35392 Giessen, Germany; 2 German Center for Lung Research (DZL), 35392 Giessen, Germany; 3 The Cardio-Pulmonary Institute (CPI), 35392 Giessen, Germany; 4 Institute for Lung Health (ILH), 35392 Giessen, Germany; 5 Department of Lung Development and Remodeling, Max Planck Institute for Heart and Lung Research, 61231 Bad Nauheim, Germany; 6 Department of Translational Pulmonology, and Translational Lung Research Center (TLRC), 69120 Heidelberg, Germany; 7 Science for Life Laboratory, Department of Molecular Biosciences, The Wenner-Gren Institute, Stockholm University, SE-10691 Stockholm, Sweden

**Keywords:** precision-cut lung slices, endocytosis, protein transport, albumin, alveolar epithelium, acute respiratory distress syndrome

## Abstract

A hallmark of acute respiratory distress syndrome (ARDS) is an accumulation of protein-rich alveolar edema that impairs gas exchange and leads to worse outcomes. Thus, understanding the mechanisms of alveolar albumin clearance is of high clinical relevance. Here, we investigated the mechanisms of the cellular albumin uptake in a three-dimensional culture of precision-cut lung slices (PCLS). We found that up to 60% of PCLS cells incorporated labeled albumin in a time- and concentration-dependent manner, whereas virtually no uptake of labeled dextran was observed. Of note, at a low temperature (4 °C), saturating albumin receptors with unlabeled albumin and an inhibition of clathrin-mediated endocytosis markedly decreased the endocytic uptake of the labeled protein, implicating a receptor-driven internalization process. Importantly, uptake rates of albumin were comparable in alveolar epithelial type I (ATI) and type II (ATII) cells, as assessed in PCLS from a SftpcCre^ERT2/+^: tdTomato^flox/flox^ mouse strain (defined as EpCAM^+^CD31^−^CD45^−^tdTomatoSPC^−^T1α^+^ for ATI and EpCAM^+^CD31^−^CD45^−^tdTomatoSPC^+^T1α^−^ for ATII cells). Once internalized, albumin was found in the early and recycling endosomes of the alveolar epithelium as well as in endothelial, mesenchymal, and hematopoietic cell populations, which might indicate transcytosis of the protein. In summary, we characterize albumin uptake in alveolar epithelial cells in the complex setting of PCLS. These findings may open new possibilities for pulmonary drug delivery that may improve the outcomes for patients with respiratory failure.

## 1. Introduction

Acute respiratory distress syndrome (ARDS) is a devastating complication of various forms of lung damage, such as viral and bacterial pneumonia or sepsis, representing a disabling condition with high mortality rates, reaching up to 46% [1]. Particularly, during the last three years, much attention has been paid to ARDS secondary to severe acute respiratory syndrome coronavirus 2 (SARS-CoV-2) infections, often leading to critical disease states [2]. One of the main hallmarks of ARDS is an increased permeability of the air–blood barrier, as a result of diffuse epithelial and endothelial damage at the distal lung, with the formation of protein-rich alveolar edema. This impairment of the alveolar-capillary barrier severely alters gas exchange and ultimately promotes deleterious alveolar remodeling [3].

Albumin, the most abundant protein in plasma, acts as a multifunctional carrier of various molecules, including hormones, vitamins, fatty acids, and pharmaceuticals [4,5]. Moreover, albumin plays a pivotal role in maintaining the oncotic pressure of the plasma, interstitial fluid, and lymph amounts. Under physiological conditions, the apical surface of the alveolar epithelium is covered by a thin fluid layer, the so-called epithelial lining fluid (ELF) [6]. The ELF contains low levels of albumin and other proteins, reaching maximal 10% of plasma concentrations. It has been previously shown that in patients with ARDS, protein levels in the alveolar edema fluid are dramatically increased. Indeed, during ARDS, alveolar protein levels are comparable to the plasma protein concentrations and are three-fold higher in non-survivors than in survivors of the disease [7,8]. Therefore, understanding the mechanisms of protein clearance from the alveolar compartment and developing new and selective therapeutic approaches to foster these processes may improve the outcomes for patients with ARDS.

Previous reports suggest that albumin is taken up by epithelial and endothelial cells by receptor-mediated endocytosis, which may be relevant for alveolar protein clearance [9,10,11,12]. However, those data have been generated in relatively simple monocultures of the above-mentioned cell types, thus lacking the aspect of the three-dimensional nature of the tissue and the potentially important interactions with other cell types of the distal lung, such as mesenchymal and hematopoietic cells. To overcome these limitations, recently, the model of precision-cut lung slices (PCLS), a viable and functional three-dimensional peripheral lung tissue, was developed [13,14]. This system enables more accurate characterization of transport processes in a model that better represents the complex structure and function of the distal lung than cellular monolayers do. Thus, in the present study, we characterized mechanisms of albumin uptake in various cell types of PCLS, primarily focusing on the alveolar epithelium. 

## 2. Results

### 2.1. Cargo-Specific Uptake of Macromolecules in Murine Precision-Cut Lung Slices

**Figure 1:**
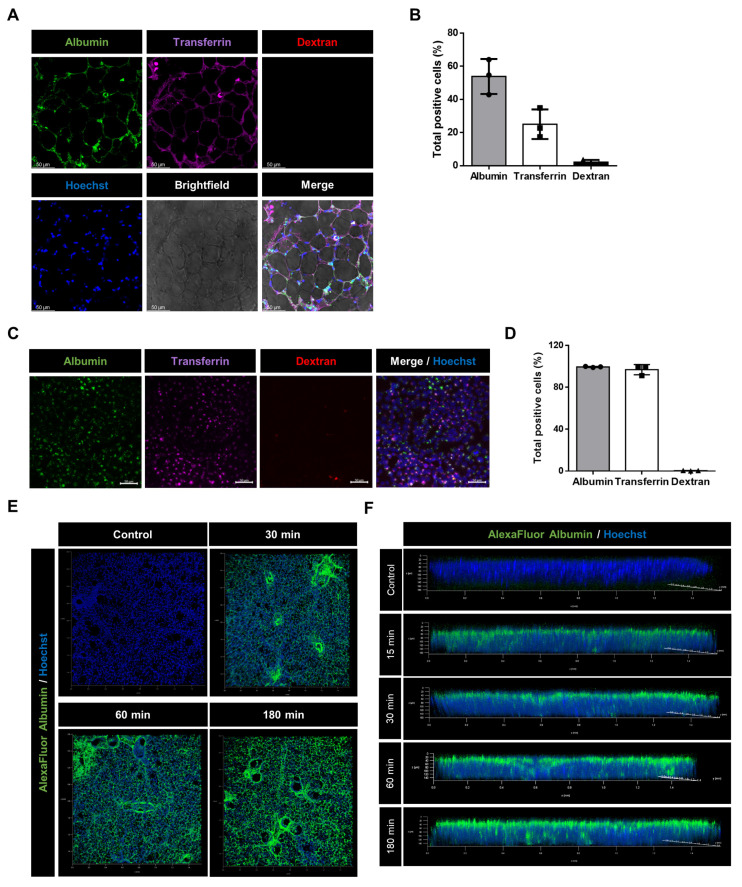
Albumin, transferrin, and dextran uptake in murine PCLS and mice MLE-12 epithelial cells: (**A**) PCLS were incubated for 1 h in a solution containing 250 μg/mL of AlexaFluor488-albumin (green), AlexaFluor647-transferrin (magenta), and lysine-fixable TexasRed-dextran (70,000 Da and red). Confocal microscopy images are shown. Scale bar−50 µM. (**B**) Flow cytometry analysis showing frequency of cells with a positive signal for each fluorochrome isolated from PCLS (expressed as a percentage). All bar graphs show mean ± SD (*n* = 3). (**C**) MLE-12 cells were incubated for 1 h in a solution containing 250 μg/mL of AlexaFluor488-albumin (green), AlexaFluor647-transferrin (purple), and lysine-fixable TexasRed-dextran (70,000 Da and red). Immunofluorescence microscopy images are shown. Scale bar−50 µM. (**D**) Flow cytometry analysis showing frequency of MLE-12 cells with a positive signal for each fluorochrome (expressed as a percentage). All bar graphs show mean ± SD (*n* = 3). (**E**,**F**) Frontal and lateral views of PCLS treated for different time intervals with AlexaFluor488-albumin (green) and stained with Hoechst (blue). Representative images are shown.

To test whether receptor-mediated endocytic transport processes play a role in the internalization of macromolecules in PCLS, we treated PCLS with different ligands: albumin, transferrin, and dextran. Albumin and transferrin were used as cargoes of receptor-mediated endocytosis and dextran as a compound to assess micropinocytosis. Next, we analyzed the amount of labeled cargoes by confocal and immunofluorescence microscopy. Our data show that both albumin and transferrin, but not dextran, were taken up by various cells of PCLS (Figure 1A). 

In a subsequent flow cytometry (FC) analysis of cells isolated from PCLS after 1 h of incubation, up to 50% of those were albumin positive and around 25% contained transferrin, whereas almost no positive signal was obtained from the dextran-treated PCLS (Figure 1B). 

These results were comparable with data generated in uptake studies performed in a cultured murine lung epithelial cell line (MLE-12) (Figure 1C,D). To further investigate the localization and distribution of internalized albumin, we treated PCLS with labeled protein and visualized it as a three-dimensional stack using confocal microscopy. As seen in the frontal and lateral views obtained from the Z-stacks in Figure 1E,F, albumin was initially predominantly localized at the apical surface and was subsequently internalized within the next 3 h.

### 2.2. Albumin Uptake in Murine Precision-Cut Lung Slices Is a Saturable Process That Depends on Endocytic Activity

**Figure 2:**
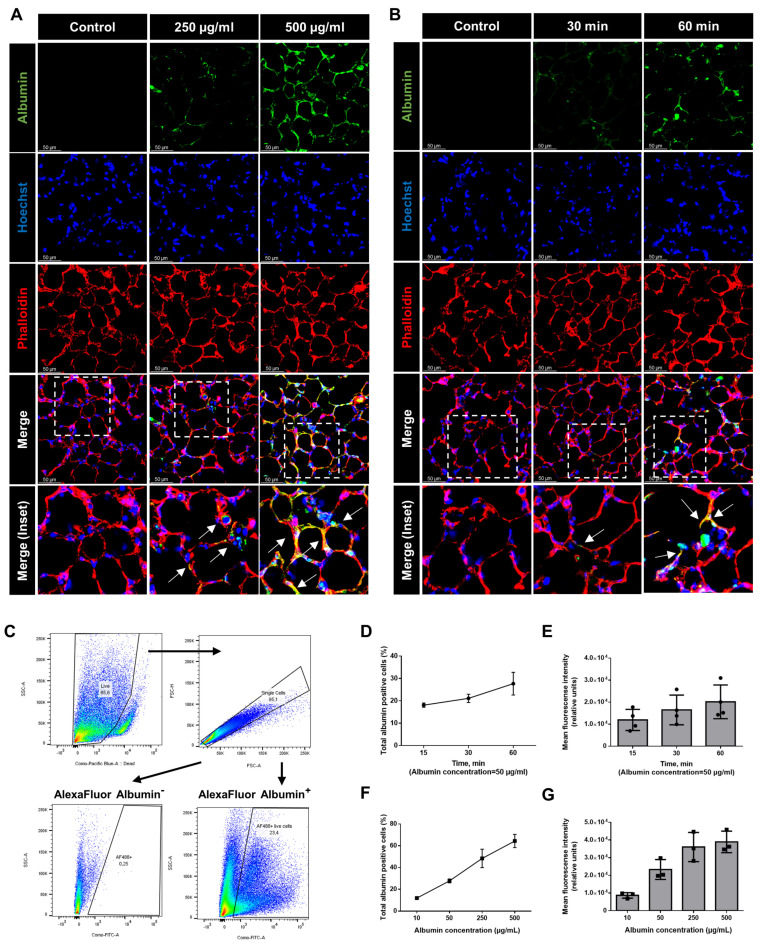
Concentration- and time-dependent uptake of albumin in PCLS: (**A**,**B**) PCLS were treated with different concentrations for up to 60 min with AlexaFluor488-albumin (green), fixed, stained with Hoechst (blue), and against phalloidin (red), and then analyzed by confocal microscopy. Representative fluorescent microscopic images are shown. The dashed line boxes mark the insets. The arrows show the co-localization of albumin and phalloidin. Scale bar−50 µM (**C**) Dot plots showing the gating strategy for isolation of albumin-positive cells. The different colors in dot plots represent the density of the fluorescent signal (**D**–**G**) Percentage and mean fluorescence intensity of albumin-positive cells isolated from PCLS at the above-mentioned concentrations and time-point. All graphs show mean ± SD (*n* = 3).

To further study the kinetics of albumin uptake in PCLS, we treated PCLS with different albumin concentrations (up to 500 µg/mL) for up to 60 min and assessed albumin internalization by immunofluorescence imaging (Figure 2A,B).

**Figure 3:**
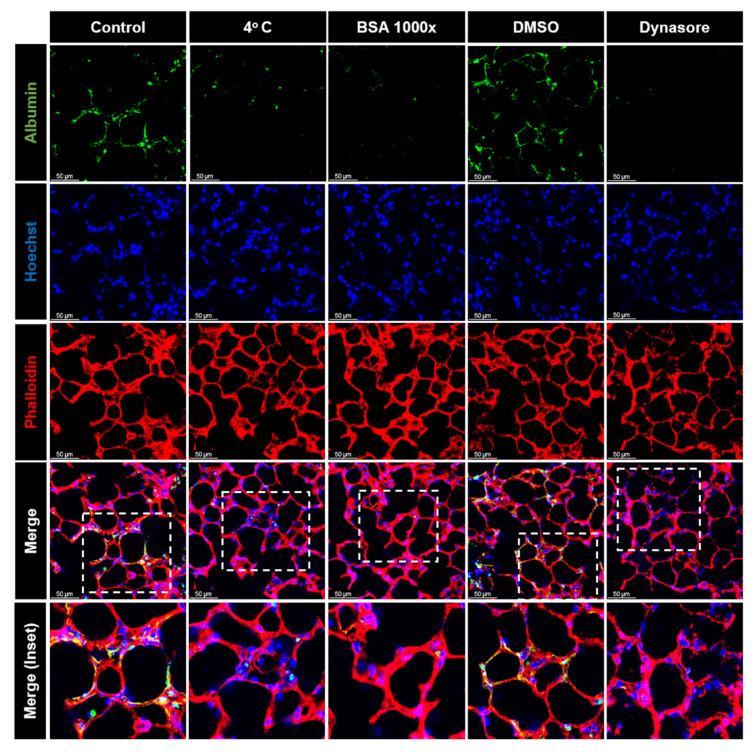
Mechanism of the albumin uptake in PCLS: murine PCLS were treated with AlexaFluor488-albumin for 60 min at 37 °C (control), 4 °C, in the presence of 1000-fold excess of bovine serum albumin (BSA), or with dynasore (or vehicle (DMSO)), and were then analyzed by confocal microscopy. Representative fluorescence images in murine PCLS are shown. Albumin (green), phalloidin (red), and nuclei (blue) are shown. The dashed line boxes mark the insets. The arrows show the co−localization of albumin and phalloidin. Scale bar−50 µM.

Indeed, we observed a time- and concentration-dependent uptake of albumin in PCLS. To further quantify these results, we also analyzed the cellular uptake by FC. The gating strategy for the detection of albumin-positive cells is depicted in Figure 2C. Our FC results confirmed that albumin uptake was time- and dose-dependent. Within the first hour by using moderate albumin concentrations (50 µg/mL), more than 30% of the cells showed a positive signal (Figure 2D,E). Moreover, by increasing the concentration of albumin to 500 µg/mL, more than 60% of the cells were found to be albumin positive (Figure 2F,G). To further investigate whether protein uptake is a saturable process, we treated PCLS with albumin at physiological 37 °C, at 4 °C, in the presence of a 1000-fold excess of native albumin, and in combination with the dynamin inhibitor, dynasore. Of note, we found that decreasing the temperature to a level where the active incorporation of macromolecules is fully inhibited, the saturation of a potential albumin receptor with non-labeled albumin, and the inhibition of receptor-mediated endocytosis in PCLS were sufficient to markedly reduce albumin uptake, as assessed by immunofluorescence imaging and FC analysis (Figure 3).

### 2.3. Alveolar Epithelial Albumin Uptake in Murine Precision-Cut Lung Slices

**Figure 4:**
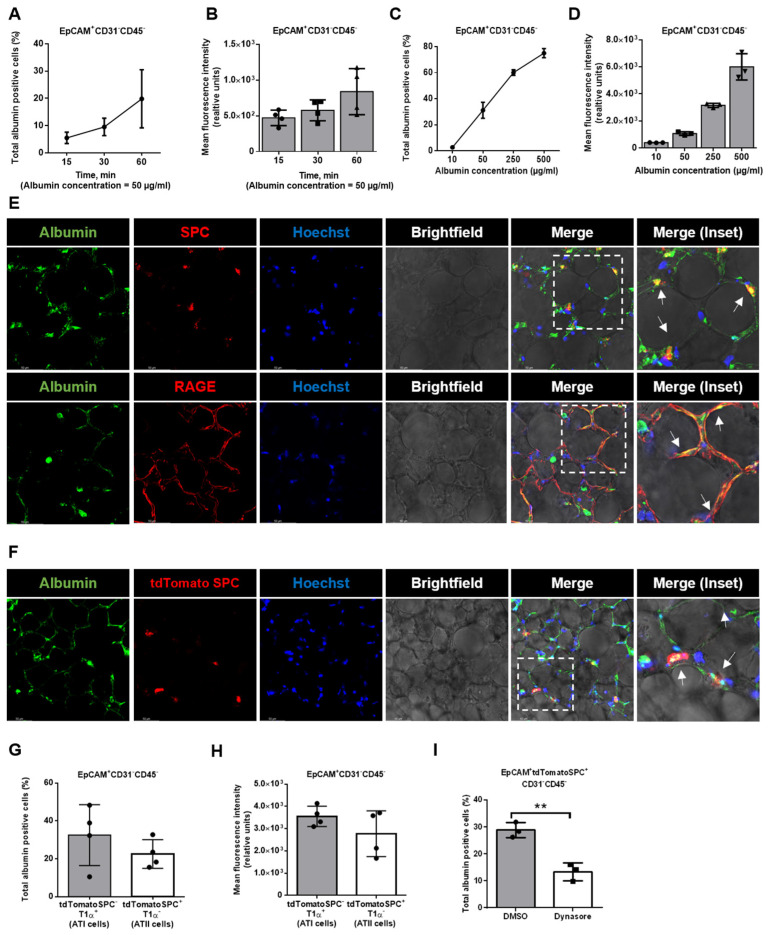
Alveolar epithelial albumin uptake in PCLS: (**A**,**C**) Murine PCLS were treated for different durations and with various concentrations of AlexaFluor488−albumin and subsequently the amount of albumin-positive epithelial cells (EpCAM^+^CD31^−^CD45^−^) was analyzed by FC (*n* = 3). (**B**,**D**) Murine PCLS were treated as described above and the mean fluorescence intensity for albumin in epithelial cells (EpCAM^+^CD31^−^CD45^−^) was analyzed by FC (*n* = 3). (**E**) Murine PCLS were treated with AlexaFluor488-albumin for 60 min and were then analyzed by confocal microscopy. Representative images of immunofluorescence staining for the ATII cell marker, SPC (red), and the ATI cell marker, RAGE (red), albumin (green), and nuclei (blue), in murine PCLS are shown. The dashed line boxes mark the insets. The arrows show the co−localization of albumin and SPC or RAGE. Scale bar−50 µM. (**F**) Murine PCLS from Sftpc^CreERT2/+^: tdTomato^flox^/^flox^ mice were treated with AlexaFluor488-albumin for 60 min and then analyzed by confocal microscopy. Representative images of immunofluorescence staining of the tomato-expressing ATII cells (red), albumin (green), and nuclei (blue) in murine PCLS are shown. The dashed line boxes mark the insets. The arrows show the co−localization of albumin and tdTomatoSPC. Scale bar−50 µM. (**G**,**H**) Murine PCLS were treated with AlexaFluor488-albumin and the percentage of albumin-positive ATI (EpCAM^+^CD31^−^CD45^−^tdTomatoSPC^−^T1α^+^) and ATII (EpCAM^+^CD31^−^CD45^−^tdTomatoSPC^+^T1α^−^) epithelial cells as well as the mean fluorescence intensity of labeled albumin were analyzed by FC. All bar graphs show mean ± SD (*n* = 4). (**I**) PCLS from Sftpc^CreERT2/+^: tdTomato^flox^/^flox^ mice were treated with AlexaFluor488-albumin in the presence or absence of an inhibitor of dynamin (dynasore), and the percentage of albumin-positive ATII (EpCAM^+^CD31^−^CD45^−^tdTomatoSPC^+^T1α^−^) epithelial cells was analyzed by FC. All bar graphs show mean ± SD (*n* = 3), ** *p* < 0.01.

We have previously studied albumin transport in cultured alveolar epithelial cell lines [10,11]. To analyze the alveolar protein transport in PCLS, a setting that better depicts the complex structure of the peripheral lung, specific cellular markers, epithelial cell adhesion molecule (EpCAM) for epithelial cells, protein tyrosine phosphatase receptor type C (CD45) for hematopoietic cells, and platelet endothelial cell adhesion molecule (CD31) for endothelial cells, were utilized. After albumin treatment, epithelial cells (EpCAM^+^CD31^−^CD45^−^) were isolated analyzed by FC. Of note, the albumin uptake in these epithelial cells was time- and dose-dependent, as assessed by treating cells with 50 µg/mL albumin for up to 1 h (Figure 4 A,B) and after the administration of various albumin concentrations (10–500 µg/mL; Figure 4 C,D). 

The alveolar epithelium consists of alveolar type I (ATI) and type II (ATII) cells. To further dissect the albumin uptake in these epithelial cell types, we performed staining against the receptor for advanced glycation end products (RAGE and ATI cells) and surfactant protein C (SPC and ATII cells). To study the contribution of ATI and ATII cells in albumin uptake, we treated PCLS with AlexaFluor488-albumin and performed confocal microscopy of the slices. As shown in Figure 4E, we detected the localization of albumin in both SPC- and RAGE-positive cells, suggesting an involvement of both alveolar cell types in albumin endocytosis in the lung.

To more precisely characterize albumin uptake in ATI and ATII cells, we next used PCLS from a Sftpc^CreERT2/+^: tdTomato^flox^/^flox^ mouse strain, in which ATII cells express the lineage trace marker, tdTomato [15]. Indeed, we confirmed the uptake of AlexaFluor488-albumin in tdTomato SPC-expressing cells by confocal immunofluorescence imaging (Figure 4F). Subsequent FC studies, in which we isolated ATI (EpCAM^+^CD31^−^CD45^−^SPC^−^tdTomatoSPC^−^T1α^+^) and ATII (EpCAM^+^CD31^−^CD45^−^SPC-tdTomatoSPC^+^T1α^−^) cells, revealed that the uptake rates of albumin in the two cell types were comparable (Figure 4G,H). In addition, and in line with our data from PCLS from wild-type mice, in treatment with the endocytosis inhibitor, dynasore markedly prevented albumin uptake in ATII cells derived from Sftpc^CreERT2/+^: tdTomato^flox^/^flox^ animals (Figure 4I).

### 2.4. Cellular Distribution of Endocytosed Albumin in Alveolar Epithelial Type II Cells in Precision-Cut Lung Slices

**Figure 5:**
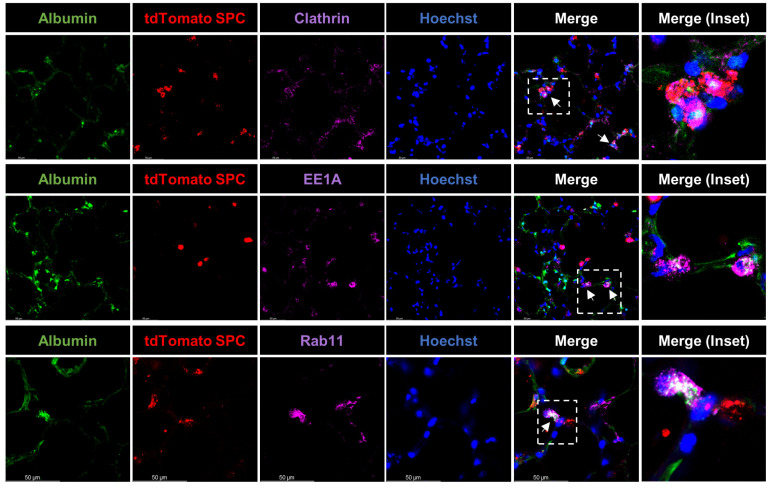
Albumin distribution upon endocytosis in PCLS: Murine PCLS from Sftpc^CreERT2/+^: tdTomato^flox^/^flox^ mice were treated with AlexaFluor488-albumin (250 µg/mL final concentration) for 60 min, fixed, and then analyzed by confocal microscopy. Representative images of immunofluorescence staining of tdTomato-surfactant protein C (SPC)-expressing ATII cells (red), clathrin, early endosome antigen 1 (EE1A, a marker of early endosomes), or Ras-related protein (Rab11, a marker of recycling endosomes) (all magenta), albumin (green), and nuclei (blue) are shown. The dashed line boxes mark the insets. Co-localization of AlexaFluor488-albumin with the above-mentioned endocytic/trafficking markers (white) is indicated by arrows. Scale bar−50 µM.

To visualize the cellular fate of the internalized albumin in ATII cells, next, PCLS derived from Sftpc^CreERT2/+^: tdTomato^flox^/^flox^ mice were treated with AlexaFluor488-albumin and stained against clathrin (receptor-mediated endocytosis), early endosome antigen 1 (EE1A, a marker of early endosomes), and Ras-related protein (Rab11, a marker of recycling endosomes; Figure 5). Our results show co-localization of labeled albumin with each of these endocytic/trafficking markers, suggesting that albumin uptake is initiated by clathrin-mediated endocytosis, followed by trafficking to the early endosomes, and subsequent exocytosis of the protein.

### 2.5. Albumin Uptake in Non-Epithelial Cell Types in Precision-Cut Lung Slices

**Figure 6:**
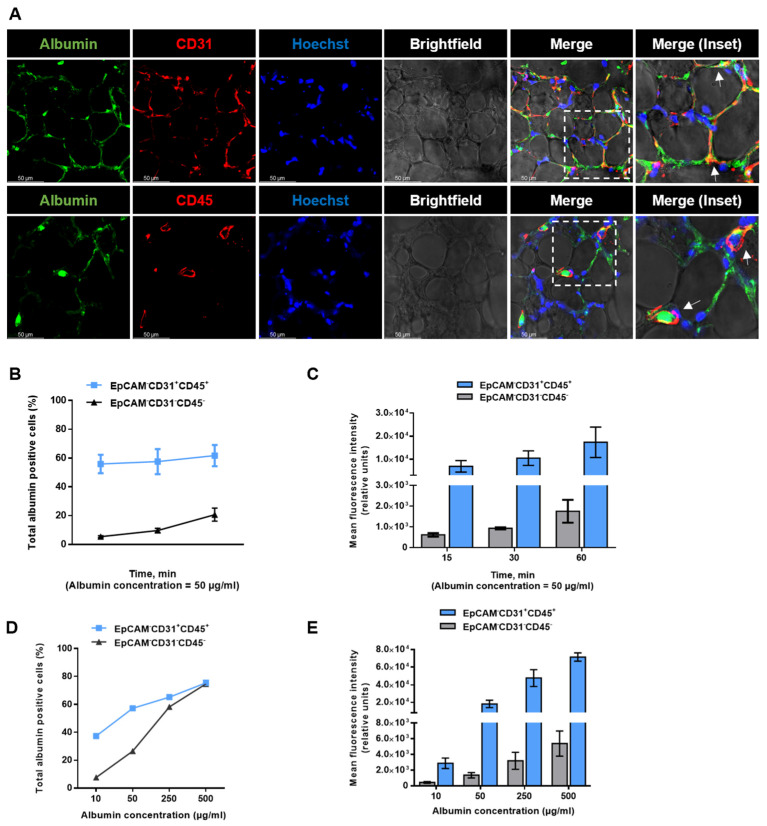
Albumin uptake in non-epithelial cell types in PCLS: (**A**) Murine PCLS were treated with AlexaFluor488-albumin for 60 min and then analyzed by confocal microscopy. Representative images of immunofluorescence staining of the endothelial cell marker, CD31, and the hematopoietic cell marker, CD45 (red), albumin (green), and nuclei (blue), in murine PCLS are depicted. The dashed line boxes mark the insets. The arrows show the co-localization of albumin and CD31 or CD45. Scale bar−50 µM. (**B**,**C**) Murine PCLS were treated with AlexaFluor488-albumin for up to 60 min, and percentage and mean fluorescent intensity of albumin-positive EpCAM^−^CD31^+^CD45^+^ and EpCAM^−^CD31^−^CD45^−^ cells were analyzed by FC. All bar graphs show mean ± SD (*n* = 4). (**D**,**E**) Murine PCLS were treated with AlexaFluor488-albumin with different concentrations of labeled albumin, and percentage and mean fluorescent intensity of albumin-positive EpCAM^−^CD31^+^CD45^+^ and EpCAM^−^CD31^−^CD45^−^ cells were analyzed by FC. All bar graphs show mean ± SD (*n* = 4).

The peripheral lung consists not only of epithelial but also of endothelial, hematopoietic, and mesenchymal cells. Therefore, we next studied the albumin uptake in non-epithelial cell types in PCLS. Of note, confocal immunofluorescence imaging of labeled albumin revealed localization in the endothelial (CD31^+^) and hematopoietic cells (CD45^+^; Figure 6A). 

To further quantify the cellular uptake of albumin in non-epithelial cells, we treated PCLS with AlexaFluor488-albumin and performed FC. As the primary focus of our study was albumin endocytosis in epithelial cells, we analyzed CD45 and CD31 cells together and characterized this cell population as EpCAM^−^CD31^+^CD45^+^, and thus, hematopoietic and endothelial cells and EpCAM^−^CD31^−^CD45^−^ represent a mesenchymal cellular population. Our results revealed a concentration- and time-dependent albumin uptake in all cellular populations studied, suggesting high uptake rates in the EpCAM^−^CD31^+^CD45^+^ cell population (Figure 6B–E). 

## 3. Discussion

In recent years, substantial progress has been made in the understanding of the pathophysiology of ARDS [1,16]. However, mortality rates in patients with this devastating syndrome remain unacceptably high, reaching 30–45% [3]. The recent and still ongoing coronavirus disease 2019 (COVID-19) pandemic dramatically increased the number of patients with acute respiratory failure due to disruptions of the alveolar-epithelial barrier, with limited therapeutic options for those patients who developed ARDS [17,18,19]. It is increasingly evident that elevated levels of proteins in the alveolar space due to disruption of the alveolar-epithelial barrier are associated with poor outcomes in patients with ARDS [3]. Therefore, uncovering the mechanisms that drive clearance of excess protein content from the alveolar space is of high clinical importance. Such studies may lead to new therapeutic approaches that by fostering transport processes across the alveolar epithelium and thus contributing to the resolution of protein-rich alveolar edema, may ultimately improve patient outcomes.

Previously, the mechanisms of albumin uptake have mostly been studied in primary and cultured alveolar epithelial cell lines [12,20,21,22]. However, such cell cultures cannot fully recapitulate the complex structure and function of the distal lung [23]. Therefore, most recently, new experimental models have been developed to study lung physiology and diseases, including alveolar liquid interface cultures, lung organoids, “organ-on-a-chip” systems, and PCLS [24,25,26]. PCLS are three-dimensional slices of the lung with a diameter of 200–500 µm that can be cultured ex vivo. PCLS may be obtained from both healthy and injured/diseased lung tissue and thus, can be used to study respiratory diseases, such as chronic obstructive pulmonary disease (COPD), asthma, lung fibrosis, and ARDS. Importantly, these preparations contain all respiratory cell types as well as the extracellular matrix and may be used to study, e.g., the effects of drugs and potential toxicity of agents, lung immunity, processes of injury, and repair and tissue remodeling [13,14,27]. In the current study, we utilized murine PCLS, as a model of viable and functional peripheral lung tissue with preserved alveolar structure, to study the endocytic processes of albumin in lung cells.

In recent years, several endocytic entry pathways have been characterized, including clathrin-mediated endocytosis (CME), caveolae-mediated endocytosis (CavME), clathrin- and caveolae-independent internalization, and micropinocytosis [28,29,30]. To characterize receptor-mediated endocytic processes in PCLS, we used two cargoes, albumin and transferrin, which have been previously shown to be rapidly internalized by CME in alveolar epithelial cells [21,31]. To test micropinocytosis, we used dextran with a molecular mass comparable to albumin (70 kDa). In line with our previous findings, we found that albumin and transferrin, but not dextran, were rapidly internalized in PCLS, similarly to cultured MLE-12 cells. Our results show that the albumin uptake in PCLS is a saturable, time- and concentration-dependent process, and the maximal velocity of which is dependent on the capacity of the endocytic machinery. These results are in line with previously published data in primary and cultured lung epithelial cells [10,21]. To further dissect the mechanisms of albumin uptake in PCLS, we used dynasore, an inhibitor of CME that specifically blocks GTPase activity of dynamin, thereby preventing the formation of clathrin-coated endocytic vesicles. Importantly, the inhibition of CME nearly completely abolished albumin uptake in PCLS, suggesting that clathrin-mediated internalization is a key regulator of albumin uptake in this setting. 

Fostering the resolution of protein-rich alveolar edema may represent a potential therapeutic means in acute lung injury [10,11]. The alveolar epithelium consists of two major cell types, ATI and ATII cells, which play different but equally important roles in maintaining optimal gas exchange and alveolar fluid balance [32]. Thus, we shifted our focus to the characterization of albumin uptake in alveolar epithelial cell types in PCLS. After confirming albumin endocytosis in both ATI and ATII cells by imaging and FC analysis, we used genetically modified mice expressing tdTomato in the SPC promoter of ATII cells to further distinguish between ATI- and ATII-specific albumin uptake and demonstrate the pivotal role of CME in albumin endocytosis. Of note, we find that the endocytosis rates in the two cell types are comparable. These results are in contrast with a previous report suggesting that protein uptake in ATII is faster than in ATI cells [20]. However, that report used ATI-like cells (differentiated from ATII cells, based on the observation that ATII cells start to express typical markers of ATI after approximately 5 days in culture) as opposed to ATI cells per se. Importantly, the setting of PCLS enables assessment of albumin endocytosis in intact, non-modified ATII and ATI cells. 

It has been previously shown that megalin/cubilin, 60-kDa glycoprotein (gp60), gp18, gp30, CD36, neonatal Fc receptor (FcRn), caveolin-1, and secreted proteins that are acidic and rich in cysteine (SPARC) may facilitate albumin uptake in various cell types [10,33,34,35,36,37]. In epithelial cells, the megalin/cubilin system is considered the main albumin receptor that initiates the CME internalization pathway [10,35,38]. In contrast, the uptake of albumin in endothelial cells is mainly orchestrated by CavME via gp60 and CD36-dependent mechanisms [34,39,40]. In hematopoietic cells (primarily macrophages in PCLS), mechanisms of albumin uptake include phagocytosis and macropinocytosis by using scavenger receptors, such as FcRn and the scavenger receptor class [41,42,43]. The detailed analyses of albumin uptake in endothelial, hematopoietic, and mesenchymal cells are beyond the scope of the current manuscript and are subjects for further research in our laboratory.

It was previously believed that the transfer of albumin across the epithelial and endothelial monolayers in alveoli was non-specific and occurred mainly through paracellular patterns. However, we and others have recently shown that in ventilated and perfused rabbit lungs, a marked proportion (approximately 30%) of aerosolized-labeled albumin, which was deposited in the alveolar compartment of the lung, was transported to the vascular compartment in an intact form within 2 h, suggesting transcytosis of the protein [10]. In line with this notion, the discovery of the above-mentioned albumin receptors in the alveolar epithelium and endothelium further highlighted the potential role of cell-specific, high-capacity, receptor-mediated endocytic processes in the context of alveolar protein clearance [10,44]. It has been shown that in epithelial cells, albumin trafficking mostly occurs via clathrin-coated vesicles [45], whereas in endothelial cells it occurs via caveolae [39]. After being internalized in alveolar epithelial cells, albumin may follow two basic pathways: exocytosis (leading to transcytosis or recycling) or degradation [30,46]. Our results obtained in tdTomato SPC-expressing ATII cells show co-localization of albumin with clathrin, the early endosome and recycling endosomes, suggesting that in ATII cells in PCLS, albumin is rapidly internalized via CME, which may be followed by transcytosis. The relatively random pattern of albumin distribution upon uptake may be explained by the fast nature of endocytic processes leading to capturing albumin fluorescence signals at different phases of cellular trafficking. However, to what extent excess albumin is endocytosed by the alveolar epithelium and subsequently degraded, recycled, or transcytosed to the interstitial space, where it might be partially cleared by lymphatics and partially be taken up by endothelial cells and eventually enter the blood (the initial source of albumin in the setting of the injured barrier), remains to be elucidated.

In summary, here we characterize albumin uptake in a fully functional, three-dimensional PCLS model. We demonstrate, for the first time, albumin uptake in this complex setting. Furthermore, we establish that albumin is internalized at comparable rates in ATI and ATII cells, which might be followed by transcytosis and thus clearance of the excess protein from the alveolar space. These findings may open new possibilities for both alveolar epithelial drug delivery and novel therapeutic approaches to improve gas exchange and, ultimately, outcomes in patients with respiratory failure.

## 4. Materials and Methods

### 4.1. Precision-Cut Lung Slices and Cell Culture

Murine PCLS were isolated from wild-type C57BL/6 mice or Sftpc^CreERT2/+^: tdTomato^flox^/^flox^ animals, as described previously [47,48]. Briefly, after filling the lungs with 0.3% agarose intratracheally, lobes were isolated and cut into 200 µm sections using a vibratome (Leica VT 1200S, Wetzlar, Germany). Isolated PCLS sections were cultured in DMEM/F12 media supplemented with 10% fetal bovine serum (FBS), 100 U/mL penicillin, 100 µg/mL streptomycin, and 1% amphotericin B. Mouse lung epithelial MLE12 cells (ATCC and CRL-2110) were obtained from the American Type Culture Collection. MLE12 cells were cultured in DMEM/F12 media supplemented with 2% fetal bovine serum, 100 U/mL penicillin, and 100 µg/mL streptomycin. Cells were kept in a humidified incubator with 5% CO_2_/95% air at 37 °C. 

### 4.2. Albumin, Transferrin, and Dextran Uptake Experiments

PCLS were incubated with 250 µg/mL (unless other specified) of AlexaFluor488-albumin, AlexaFluor647-transferrin, and TexasRed-dextran (Thermo Scientific, Eugene, OR, USA), and dissolved in DPBS (PAN Biotech, Aidenbach, Germany) for 1 h. At the end of the experiment, cultures were rinsed three times with ice-cold PBS and then subjected to immunofluorescence or FC.

### 4.3. Flow Cytometry

Cultured and primary epithelial cells were lifted to form culture dishes by incubating with trypsin 1X (Thermo Fisher Scientific, Darmstadt, Germany). PCLS were digested for 20 h at 37 °C with a solution containing elastase 250 ng/mL (Elastin Products Co. Inc, Owensville, MO, USA) and trypsin 1X. The cells were pelleted at 350 g for 10 min at 4 °C, blocked with a pooled immunoglobulin G (IgG) antibody preparation, and stained for 15 min at 4 °C in MACS buffer (PBS, 7.4% EDTA, 0.5% FBS pH 7.2). Next, the cells were stained with allophycocyanin (APC)-conjugated antibodies. The following were used: Cy7 anti-mouse EpCAM (Biolegend, San Diego, CA, USA), APC anti-mouse Podoplanin (Biolegend, San Diego, CA, USA), PE-CF594 rat anti-mouse CD31 (BD Horizon, Heidelberg, Germany), PE-CF594 rat anti-mouse CD45 (BD Horizon, Heidelberg, Germany), and Zombie Violet Fixable Viability Kit (Biolegend, San Diego, CA, USA). Cells were then washed once with MACS buffer, pelleted, and resuspended in 200 μL of MACS buffer before being filtered into a FC tube for cell analysis. Multicolor flow cytometry was performed on a BD FACS Fortessa III using DIVA software (BD Bioscience, Heidelberg, Germany). Gating strategy used to identify mouse ATII cells is shown in Figure 1. After excluding doublets and dead cells, followed by exclusion of endothelial CD31^+^ and cells of hematopoietic origin identified by CD45^+^, ATII cells were identified as EpCAM^+^CD45^−^CD31^−^tdTomato-SPC^+^ cells.

### 4.4. Immunofluorescence and Confocal Imaging

PCLS or MLE12 cells were incubated in eight-well chamber slides (Ibidi, Gräfelfing, Germany). After incubation with albumin, transferrin, or dextran, cells were washed and fixed in 4% paraformaldehyde (Sigma-Aldrich, St. Louis, MO, USA). After fixation, cells were permeabilized with 0.01% Triton X-100 for 5 min. After overnight incubation with primary antibodies at 4 °C, secondary antibodies were added for 1 h and stained with Hoechst (Thermo Scientific, Eugene, OR, USA) for 30 min. Fluorescence was captured by using Leica TCS SP5 (Leica Microsystems, Wetzlar, Germany) or Carl Zeiss Axio+ immunofluorescence microscope (Carl Zeiss, Wetzlar, Germany). 

### 4.5. Antibodies and Reagents

Dynasore was obtained from Sigma-Aldrich. AlexaFluor 488-albumin from bovine serum conjugate, TexasRed-dextran (70,000 MW) lysine fixable, and AlexaFluor594-transferrin were from ThermoFisher Scientific. AlexaFluor594-phalloidin was obtained from ThermoFisher Scientific. The following antibodies and reagents were used: rabbit anti-prosurfactant protein C (Abcam, Cambridge, UK), rat anti-RAGE (R&D Systems, Minneapolis, MN, USA), rabbit anti-EEA1, anti-Rab11, and anti-clathrin from Cell Signaling (Cell Signaling, Danvers, MA, USA). 

### 4.6. Statistical Analysis

Data are presented as mean ± SD and were analyzed using one-or two-way analysis of variance (ANOVA) for multiple comparisons using GrapdPad Prism version 6.0 (GraphPad Software). *p*-values of <0.05 were considered to be statistically significant. 

## Data Availability

Not applicable.
